# Impact of local terminology on the design of a community-based diagnostic and management algorithm for postpartum sepsis: findings of formative research

**DOI:** 10.1093/inthealth/ihab030

**Published:** 2021-05-27

**Authors:** Shabina Ariff, Fatima Mir, Wafa Aftab, Farrukh Raza, Shujaat Zaidi, Sheraz Memon, Amnesty LeFevre, Linda A Bartlett, Peter Winch, Sajid Bashir Soofi, Zulfiqar A Bhutta

**Affiliations:** Department of Paediatrics and Child Health, Aga Khan University, Karachi, Pakistan; Department of Paediatrics and Child Health, Aga Khan University, Karachi, Pakistan; Department of Paediatrics and Child Health, Aga Khan University, Karachi, Pakistan; Center of Excellence in Women & Child Health, Aga Khan University, Karachi, Pakistan; Center of Excellence in Women & Child Health, Aga Khan University, Karachi, Pakistan; Department of Paediatrics and Child Health, Aga Khan University, Karachi, Pakistan; Department of International Health, Johns Hopkins Bloomberg School of Public Health, Baltimore, MD, USA; Department of International Health, Johns Hopkins Bloomberg School of Public Health, Baltimore, MD, USA; Department of International Health, Johns Hopkins Bloomberg School of Public Health, Baltimore, MD, USA; Department of Paediatrics and Child Health, Aga Khan University, Karachi, Pakistan; Center of Excellence in Women & Child Health, Aga Khan University, Karachi, Pakistan; Center of Excellence in Women & Child Health, Aga Khan University, Karachi, Pakistan

**Keywords:** local, management algorithm, maternal health, puerperal sepsis, terminology

## Abstract

**Background:**

Postpartum sepsis is one of the leading causes of maternal mortality and morbidity in developing countries. This formative research elicits local terms used for postpartum illnesses and symptoms of postpartum sepsis with the aim of improving postpartum diagnosis and management in Pakistan.

**Methods:**

We conducted 34 in-depth interviews with recently delivered women (RDW), traditional birth attendants (TBAs), healthcare providers and family members of RDW from rural Sindh to explore local Sindhi terms used to describe postpartum sepsis and related symptoms. During interviews, all participants were asked to orally free list common symptoms of postpartum illnesses; those who were aware of the concept were asked to free list possible symptoms of postpartum sepsis. The responses were recorded by the interviewer. Free listing data were analyzed for frequency and salience.

**Results:**

Most participants, including TBAs, were not familiar with the concept of postpartum sepsis as a distinct disease or of a local term denoting the concept. Almost all could identify and report symptoms related to postpartum sepsis in the local language. Only physicians were able to recognize the term postpartum sepsis and related symptoms. Multiple local terms were used for a particular symptom; still others were used to denote gradations of severity. ‘Bukhar’ (fever) was the most commonly named symptom although it was often considered a normal part of puerperium. Many postpartum illnesses were related to the highly non-specific local term ‘kamzori’ (weakness).

**Conclusions:**

Better awareness about local terminology used in rural areas related to postpartum sepsis could improve communication, care-seeking patterns, diagnosis and management.

## Introduction

In 2008, global estimates indicated that there were over 343 000 maternal deaths. It is estimated that 26 000 maternal deaths occurred in Pakistan during 2000, making it the third highest country for maternal deaths, after India and Nigeria. Additionally, it is 1 of 13 countries that contribute 67% of all maternal deaths.^1^ Maternal postpartum sepsis is the third leading cause of maternal mortality in the world, accounting for 12% of all global maternal deaths.^2^

The WHO defines postpartum sepsis as an ‘infection of the genital tract occurring at any time between the onset of the rupture of membranes or labor and the 42nd day postpartum in which fever and one or more of the following are present: pelvic pain, abnormal vaginal discharge, abnormal odor of discharge, and delay in the rate of reduction of size of the uterus’.^3^ In developing countries, postpartum infections are most often acquired through unhygienic delivery or by organisms from the lower genital tract.^4,5^ Among those who survive, morbidity rates are high and include injuries, infections, debilitating disabilities and infertility. Infections may also be transmitted to newborns antepartum or during the delivery.^6,7^

A number of barriers hamper women's access to appropriate healthcare, including cultural norms forbidding women from traveling without their family's permission, financial constraints and transportation issues, as well as local beliefs about health and wellness.^8^ Seeking remedies from traditional healers and the lower priority given to women's health in many South Asian cultures are also factors limiting access to appropriate healthcare among women.^9,10^ Furthermore, studies have shown that a number of potentially dangerous signs, such as abnormal vaginal bleeding, are considered a normal part of puerperium, leading to delayed care-seeking.^11^ A low level of knowledge about danger signs in the postpartum period among Pakistani women (2–22%) and men (1–15%) is also a contributory factor.^12^

Previous research has shown that the use of various colloquial terms among rural South Asian women deeply affects their perception of diseases and care-seeking practices.^13^ Additionally, the use of biomedical terms in the community do not always correspond to their actual definitions, thereby creating confusion among healthcare providers.^14^ There is limited research available on the local terminology used for postpartum sepsis in Pakistan. This study aims to describe the local terminology related to postpartum sepsis in a rural community in Sindh, Pakistan.

## Methods

### Study site and participants

This formative study was conducted in Matiari, Pakistan. Matiari is a rural district in the province of Sindh. It has 1400 villages and an official population of 0.6 million. The annual expected birth rate of this population is 25 per 1000. Study participants were randomly selected from other study databases. We purposively selected 34 participants (recently delivered women [RDW], senior family members of RDW, traditional birth attendants [TBAs], physicians and other healthcare providers) for in-depth interviews. Interviews were conducted in the community and at district headquarter hospital, Matiari. The participants were not provided with monetary incentives, but the study staff arranged refreshments such as biscuits and juice. A total of 7 RDW within 42 d of delivery, 6 family members of RDW and 8 TBAs were interviewed within the community. At the facility site, 4 women diagnosed with postpartum sepsis and admitted for treatment, 3 family members of RDW, as well as 6 physicians with experience in diagnosing postpartum sepsis, were interviewed.

### Data collection

Verbal informed consent was obtained from all participants before conducting the interviews. Each interview lasted 30–45 min. We developed semistructured in-depth interview guides with major questions, subquestions and probes in English and these were translated into the local language. Trained moderators conducted all the interviews in Sindhi, the main spoken language in the study district. During the interviews, a moderator, notetaker and observer were present as per study protocol. The interviews explored various postpartum symptoms, terminologies used for symptoms in the local language and knowledge about postpartum sepsis through free listing. Free listing is a systematic data collection method where an informant is asked to make a list of all the different kinds of a category.^15^

### Data analysis

The interview notes were transcribed into a computer and analyzed for frequency scores and salience scores. Frequency scores identify how commonly a term is used by participants. Salience scores are derived from the frequency, the order in which a term was mentioned and the length of the list.^15,16^ NVivo software was used for data analysis. All responses were coded then emerging themes and subthemes were analyzed.

## Results

A total of 34 interviews were conducted, 13 at the health facility and 21 within the community. Overall, most participants were aged 18–35 y, illiterate and identified themselves as housewives. Physicians were the only participants with a postsecondary education.

### Recognition of postpartum sepsis

The findings suggest that almost all participants were unaware of postpartum sepsis and its local terminology. Even TBAs, who are commonly involved in peripartum care and births, were unaware of postpartum sepsis or any related term in Sindhi. Only physicians were aware of postpartum sepsis and related local terminology. In terms of symptoms, relatively senior physicians were better able to recognize postpartum sepsis. Referring to the symptoms and signs associated with postpartum sepsis, one physician (P) said:

*Breathing becomes fast, there is fever, dehydration, uterus will not decrease in size as much as it should, there is pain in the abdomen below the umbilicus, and there is foul smelling discharge. These are the common symptoms* (P1).

Most participants were comfortable talking about reproductive and postpartum symptoms. Many could name and describe various symptoms indicative of postpartum sepsis. However, none of them, except for the physicians, considered these symptoms to be suggestive of postpartum sepsis.

### Local terminology for fever

Of the postpartum symptoms, fever was most commonly mentioned as ‘bukhar’ in Sindhi. In the free listing exercise, it was usually one of the first terms listed by respondents, with a frequency score of 79.4. It was also the most salient term, with a salience score of 0.611. Table 1 provides the salience and frequency scores of postpartum sepsis symptoms in Sindhi.

**Table 1.** Salience and frequency of postpartum sepsis symptom terms in Sindhi

Symptom	Sindhi term	Frequency^a^ (N=34)	Salience^b^ (N=34)	Meaning and common usage
Fever	Bukhar	79.4	0.611	‘Bukhar’ is a commonly used term for fever. It is combined with various adjectives such as ‘sano’ (low), ‘sayo’ (high), ‘tez’ (high) to depict severity
Pelvic pain	Naran mai soor	64.7	0.484	Meaning pain in pelvic area. The term ‘naran’ refers to the lower part of the abdomen. It is commonly used for pain related to female reproductive system problems. ‘Soor’ means pain
Bleeding	Khoon halan	32.4	0.213	Means losing blood
Abdominal pain	Pait mai soor	32. 4	0.179	Means pain in the abdomen. The word ‘pait’ can denote either abdomen or stomach in Sindhi. When used in connection with pain it generally means pain anywhere in the abdomen
Vaginal discharge	Hethiyan pani achan	23.5	0.116	Literally means water coming from below. The term ‘pani’ means water and can be combined with various adjectives to denote qualities such as ‘dhap varo pani’, which literally means water with bad smell or ‘gando pani’, which means dirty water

a Frequency shows how commonly the term was mentioned by the participants during free listing. Higher scores show more frequent use.

b Salience is a combined index of the frequency with which the term is used and the order in which it is mentioned in terms of importance. It shows how important participants consider a symptom to be. A higher score means higher salience.

Additionally, participants used a number of other local terms for fever and its levels of severity. ‘Tapp’ was another commonly used term for fever. The presence of a high-grade fever was denoted by the term ‘seyo bukhar’. Although this term translates into fever with chills, it was used to indicate a high-grade fever. Other terms used to identify a high-grade fever were ‘Tez bukhar’ and ‘Bukhar jam aahey’. ‘Bukhar jam aahey’ translates into a lot of fever. ‘Hadd mai bukhar’ and ‘sano sano bukhar’ were both used to indicate a low-grade fever. Alternative local terms for various symptoms are specified in Table 2.

**Table 2.** Alternative terms for the most commonly used Sindhi postpartum sepsis symptoms

Symptom	Most commonly used Sindhi term	Alternative terms	Terms for varying severity
Fever	Bukhar (fever)	Tapp (fever)	Seyo bukhar (fever with chills)
			Tez bukhar (high grade fever)
			Bukhar jam aahey (a lot of fever)
			Hadd mai bukhar (low grade fever)
			Sano sano bukhar (slight fever)
Pelvic pain	Naran mai soor (pain in pelvic area)	Nanran mai takleef (pain in pelvic area)	-
		Bachadani mai soor (pain in uterus)	
Bleeding	Khoon halan (bleeding)	Khoon (blood)	-
Abdominal pain	Pait mai soor (pain in abdomen)	Pait ji takleef (pain in abdomen)	-
Vaginal discharge	Hethiyan pani achan (water from below)	Pani (discharge)	Badbu varo pani (foul-smelling discharge)
		Paidaish ware raste ma pani (discharge from birth canal)	Dhap varo pani (foul-smelling discharge)
			Gando pani (dirty discharge)

Interestingly, most participants considered fever to be a normal part of puerperium and recovery from the childbirth process. The presence of fever on its own was not considered an important enough symptom to encourage women and their families to seek help from a healthcare provider or TBA. ‘Hadd mai bukhar’ means limited fever, which by itself carries connotations of a problem that is under control and does not need serious attention. ‘Sano sano bukhar’ (low, low, fever), along with ‘had mai bukhar’, were considered to be part of puerperium. Some women did regard ‘seyo bukhar’ and ‘tez bukhar’ (high-grade fever) as important symptoms, but not serious enough to take precedence over household duties. One woman, who had been diagnosed with postpartum sepsis, said:

*I had ‘bukhar jam aahey’* [high-grade fever], *which was not resolving but where could my children go, so I didn't go to take medicine* (RDW1).

Healthcare providers, however, considered persistent fever as an important sign on its own to prompt proper medical treatment. For instance, one physician talking about what a woman's family should do regarding seeking care, said:

*They should get good treatment quickly. If they still feel that ‘bukhar’ is not settling, then she should be taken to a big hospital* (P2).

Physicians also emphasized the severity of the fever as an important aspect for care-seeking. Fever combined with other symptoms was recognized as an indicator of a potentially serious illness by both women and physicians. According to one physician,

*[The woman] who has high grade fever more than 101°F, who has pelvic infection, swollen abdomen and vomiting, she should be given treatment urgently* (P3).

### Local terminology for reproductive health symptoms

Among other reproductive health symptoms, ‘naran mai soor’ (pelvic pain) and ‘khoon halan’ (bleeding) were terms with a high salience (Table 1). Unlike fever, pelvic pain was regarded as an important symptom to prompt care-seeking. According to one RDW,

*TBA was not called for childbirth but TBA was called when she had ‘naran mai soor’* [pain in the pelvic area] (RDW2).

‘Hethiyan pani achan’ (vaginal discharge) was also an important symptom of postpartum sepsis. According to one physician,

*‘Dhap varo pani’* [foul-smelling vaginal discharge] *could be another sign of postpartum sepsis* (P4).

Among participants, vaginal discharge had a higher number of alternative terms than any other symptom. The terms ranged from the innocuous sounding ‘pani’ (water) to the more descriptive terms ‘paidaish ware raste mai pani’ (discharge from the birth canal) and ‘dhap varo pani’ (foul-smelling discharge). The many terms used for vaginal discharge may be responsible for the low salience (0.116) and frequency (23.5) scores identified for this symptom.

### Local terminology for other symptoms

‘Kamzori’ (low energy or weakness) was a frequently mentioned symptom of postpartum illness. Rather than being a single disease symptom, kamzori was used to denote a constellation of symptoms representing a state of feeling unwell. According to participants, the signs of kamzori included body aches, a lack of energy, tiredness and a lack of interest or inability to perform household tasks. In our study, kamzori was considered common but unlikely to lead to serious or fatal outcomes. Kamzori was considered to be a sign and cause of many health problems during the postpartum period among the study population.

‘Bukh na lagdi aahey’ (lack of appetite) was also considered a common symptom associated with postpartum illness. A number of participants related the severity of the patient's condition to the severity of body aches and pains. In cases where women were unable to carry out household tasks, they were considered to be seriously ill and were labeled as ‘soor je karey thi wehi kona saghan’ (cannot get up due to severe pain). In that sense, the seriousness of a disease’s process is correlated to a woman's ability to perform everyday tasks, rather than to a particular symptom. For instance, one RDW said:

*Who will do the [house] work and who will look after the kids if I don't feel well? If I feel sick, I go to take medicine so I can get better and do the work* (RDW3).

Making a similar point, another RDW said:

*I had high grade fever. My poor kids didn't know what to do. If I wasn't worried about them, I would not have taken medication* (RDW5).

‘Khoon ji kami’, a lack of blood or anemia, was also considered an important symptom among postpartum women. According to one healthcare provider,

*[I]f the woman has deficiency of blood, it could cause a lot of health problems* (TBA1).

However, in the local parlance, ‘khoon ji kami’ does not necessarily convey the biomedical concept of anemia. It could refer to either general pallor or even generalized weakness. In that sense, it is referred to as an underlying cause of weakness, rather than as a sign by itself.

## Discussion

Our findings suggest that knowledge of postpartum sepsis is lacking among the residents of rural Sindhi communities. This lack of knowledge may be the result of there being no existing local term for postpartum sepsis. RDW, family members of RDW and TBAs could list and describe clinical symptoms of postpartum sepsis, but they could not relate the collection of symptoms to a serious disease process.

The overall inability of community members and healthcare providers to relate symptoms to postpartum sepsis may significantly delay care-seeking. Furthermore, the inherent hierarchy of postpartum sepsis symptoms present within the community may also delay care-seeking by women. Within the community, symptoms such as fever were seen as less serious than foul-smelling vaginal discharge.

The large number of local terms used to refer to one symptom may reflect the reserve and reticence of the local culture regarding reproductive health symptoms. Thus, the need to balance modest ambiguity with necessary description might be behind the multiple terms for postpartum sepsis. These cultural impediments make it difficult to suggest one specific term for a specific symptom.

‘Kamzori’ is another local term with a broad constellation of symptoms and different nuances in different circumstances. However, rather than referring to a specific disease or complex symptoms, kamzori is akin to an idiom of distress used to denote many states of ill health. Similar to our findings, a variant term for kamzori labeled ‘kamjori’ is used by rural Indian women to refer to a condition that causes physical weakness, palpitations, loss of appetite, lack of interest in day-to-day activities and blackouts. In addition to these symptoms, kamjori also expresses mental fatigue, depression and loss of sexual interest.^14^ Contrary to our findings, kamjori was considered a potentially fatal condition among Indian women. It was also considered a symptom and cause of ill health; this was evident among other South Asian cultures. In Nepal, ‘kamjori’ refers to an underlying cause of postpartum illnesses such as abnormal postpartum bleeding.^17^

The ability of a woman to perform her household tasks is used as a measure of her health status in rural Sindh. The fact that people describe the seriousness of a disease in functional terms has implications for care-seeking practices and communication. Women and their families are more inclined to seek care if a woman cannot perform her household chores. Another possibility could be that women find it easier to justify the extra expense and time needed for treatment by expressing their health status in functional terms. This tendency to relate women's health to their functional ability has been noted among women in India.^11^ Research identified that women themselves downplay their symptoms and relate them to functional ability, which could be because of their cultural conditioning, which accustoms them to ascribe less importance to women's health problems. Thus, communication with families about women's health in terms of their ability to contribute to household functions might be more meaningful for accessing timely care.

To improve care-seeking among RDW, healthcare providers must increase their knowledge of the local terminology used for postpartum illnesses. For example, in rural South Asia, terms such as kamzori are central to women's understanding and expression of disease processes, and without incorporating them in clinical discussions, physicians may find it difficult to gain a patient's trust.^14^ Additionally, improved knowledge of local terminology and cultural beliefs may enable healthcare providers to communicate easily and prescribe the appropriate treatment for participants.

Furthermore, the findings of the current study provide sufficient evidence regarding the local terminology of postpartum sepsis to develop a community-based educational program for RDW and healthcare providers. By improving community awareness of postpartum sepsis using local terminology, there may be an increase in appropriate symptom recognition, timely referral to healthcare providers and prompt care-seeking practices among women.

### Limitations and challenges

To our knowledge, this is the first study to explore the local terminology related to postpartum sepsis in rural Pakistan. RDW, TBAs and family members of RDW were forthcoming when discussing their knowledge and viewpoints of symptoms associated with postpartum sepsis. Furthermore, physicians were open about the challenges and difficulties faced when identifying local terminology related to symptoms of postpartum sepsis. Our research has identified important local terminology, drawing attention to the importance of educating healthcare providers and the community about the need to correctly recognize symptoms and seek care promptly. Participants were recruited from one local hospital and within one community, therefore the local terms expressed may not represent the terminology used across Pakistan or in other South Asian countries.

### Conclusion

Awareness of postpartum sepsis and related symptoms was limited among RDW, family members of RDW and community healthcare providers; however, physicians were found to be knowledgeable regarding the disease and its symptoms. Based on the in-depth interviews, the local terms used to describe postpartum sepsis and symptoms were bukhar (fever), naran mai soor (pelvic pain), khoon halan (bleeding), pait mai soor (abdominal pain) and hethiyan pani achan (vaginal discharge). The most frequent and salient term was bukhar, followed by naran mai soor, khoon halan, pait mai soor and hethiyan pani achan. The local terminology identified through our study will help in developing appropriate community-based educational programs to improve care-seeking practices, communication and trust among physicians and the community.

## Data Availability

Data can be made available upon request to the corresponding author.
